# De Novo Genome Assembly Highlights the Role of Lineage-Specific Gene Duplications in the Evolution of Venom in Fea's Viper (*Azemiops feae*)

**DOI:** 10.1093/gbe/evac082

**Published:** 2022-06-07

**Authors:** Edward A Myers, Jason L Strickland, Rhett M Rautsaw, Andrew J Mason, Tristan D Schramer, Gunnar S Nystrom, Michael P Hogan, Shibu Yooseph, Darin R Rokyta, Christopher L Parkinson

**Affiliations:** Department of Biological Sciences, Clemson University, Clemson, SC 29634, USA; Department of Biological Sciences, Clemson University, Clemson, SC 29634, USA; Department of Biology, University of South Alabama, Mobile, AL 36688, USA; Department of Biological Sciences, Clemson University, Clemson, SC 29634, USA; Department of Biological Sciences, Clemson University, Clemson, SC 29634, USA; Department of Evolution, Ecology and Organismal Biology, The Ohio State University, Columbus, OH 43210, USA; Department of Biological Sciences, Clemson University, Clemson, SC 29634, USA; Department of Biological Science, Florida State University, Tallahassee, FL 32306, USA; Department of Biological Science, Florida State University, Tallahassee, FL 32306, USA; Department of Computer Science, Genomics and Bioinformatics Cluster, University of Central Florida, 4000 Central Florida Blvd, Orlando, FL 32816, USA; Department of Biological Science, Florida State University, Tallahassee, FL 32306, USA; Department of Biological Sciences, Clemson University, Clemson, SC 29634, USA; Department of Forestry and Environmental Conservation, Clemson University, Clemson, SC 29634, USA

**Keywords:** snake genomics, Viperidae, venom evolution, gene family expansion

## Abstract

Despite the medical significance to humans and important ecological roles filled by vipers, few high-quality genomic resources exist for these snakes outside of a few genera of pitvipers. Here we sequence, assemble, and annotate the genome of Fea’s Viper (*Azemiops feae*). This taxon is distributed in East Asia and belongs to a monotypic subfamily, sister to the pitvipers. The newly sequenced genome resulted in a 1.56 Gb assembly, a contig N50 of 1.59 Mb, with 97.6% of the genome assembly in contigs >50 Kb, and a BUSCO completeness of 92.4%. We found that *A. feae* venom is primarily composed of phospholipase A_2_ (PLA_2_) proteins expressed by genes that likely arose from lineage-specific PLA_2_ gene duplications. Additionally, we show that renin, an enzyme associated with blood pressure regulation in mammals and known from the venoms of two viper species including *A. feae*, is expressed in the venom gland at comparative levels to known toxins and is present in the venom proteome. The cooption of this gene as a toxin may be more widespread in viperids than currently known. To investigate the historical population demographics of *A. feae*, we performed coalescent-based analyses and determined that the effective population size has remained stable over the last 100 kyr. This suggests Quaternary glacial cycles likely had minimal influence on the demographic history of *A. feae*. This newly assembled genome will be an important resource for studying the genomic basis of phenotypic evolution and understanding the diversification of venom toxin gene families.

**Significance**

We provide the first de novo genome assembly for Fea’s Viper (*Azemiops feae*). This genome was assembled with PacBio continuous long read data, polished using Illumina short-read data, and annotated with transcriptome data from numerous tissues. Using venom gland transcriptomics and the venom proteome, we ascertain the expression profile of toxins and evaluate the evolution of the phospholipase A_2_ gene family, one of the most abundant and functionally diverse toxins in Viperidae. These genomic resources will be valuable for better understanding the genomic basis of complex traits, investigating the origins and diversification of the highly diverse and successful radiation of Viperids, and exploring community-wide patterns of historical demography in East Asia throughout the Quaternary.

## Introduction

Snakes in the family, Viperidae, are a diverse group of venomous snakes that have received significant investigation, particularly in terms of life histories, systematics, and venom composition (Lynch 2007; Hendry et al. 2014; Alencar et al. 2016). For example, this clade contains both viviparous and oviparous species and many taxa exhibit parental care behaviors (e.g., Greene et al. 2002). Body sizes of viperids span over an order of magnitude, from a maximum body length of 375 cm (*Lachesis muta*) to ≤ 28 cm (*Bitis schnederi*; Vitt and Caldwell 2013). Viperids are nearly globally distributed, span a wide latitudinal range, from 45°S latitude in Argentina to 66.5°N above the Arctic circle in Scandinavia (Vitt and Caldwell 2013), and they occur in habitats ranging from sea level to high elevation montane forests and from deserts to tropical rainforests. Finally, viperids display extensive variation in venom from highly neurotoxic venoms to hemorrhagic venoms within single genera (Mackessy 2010). The extensive ecological, morphological, and physiological diversity found across viperids provides an exemplary system for comparative analyses.

Understanding evolutionary history and the genetic basis of species-specific traits has been accelerated by the proliferation of whole genome assemblies in many taxa (Feng et al. 2020; Kim et al. 2021). Genomic sequencing efforts within viperids thus far have focused on the pitvipers (Crotalinae), largely within *Crotalus* (five species sequenced to date) and *Protobothrops* (two species sequenced), to understand the evolution of venom and sensory biology (Gilbert et al. 2014; Aird et al. 2015; Shibata et al. 2018; Schield et al. 2019; Hogan et al. 2021; Margres et al. 2021). To fully understand the genetic mechanisms driving phenotypic evolution and species diversification, additional genomic resources are needed across the viperid tree of life.

Here, we sequence the genome of a Fea’s Viper, *A. feae*, an enigmatic viperid species representative of the monotypic subfamily Azemiopinae (but see Nikolai et al. 2013; Li et al. 2020). This taxon is sister to the Crotalinae, a successful radiation of ∼230 species, which currently accounts for nearly all genomic resources available for vipers. With this first full-genome assembly of *A. feae*, we investigate the origins of venom components with a focus on the phospholipase A_2_ (PLA_2_) gene family and the expression of renin in the venom gland, which has been previously identified in *Azemiops* and *Echis* venom. Furthermore, we explore the demographic history of *A. feae* and discuss this in comparison to codistributed species.

## Results and Discussion

### Genome Assembly and Structural Contents

**Fig. 1.**
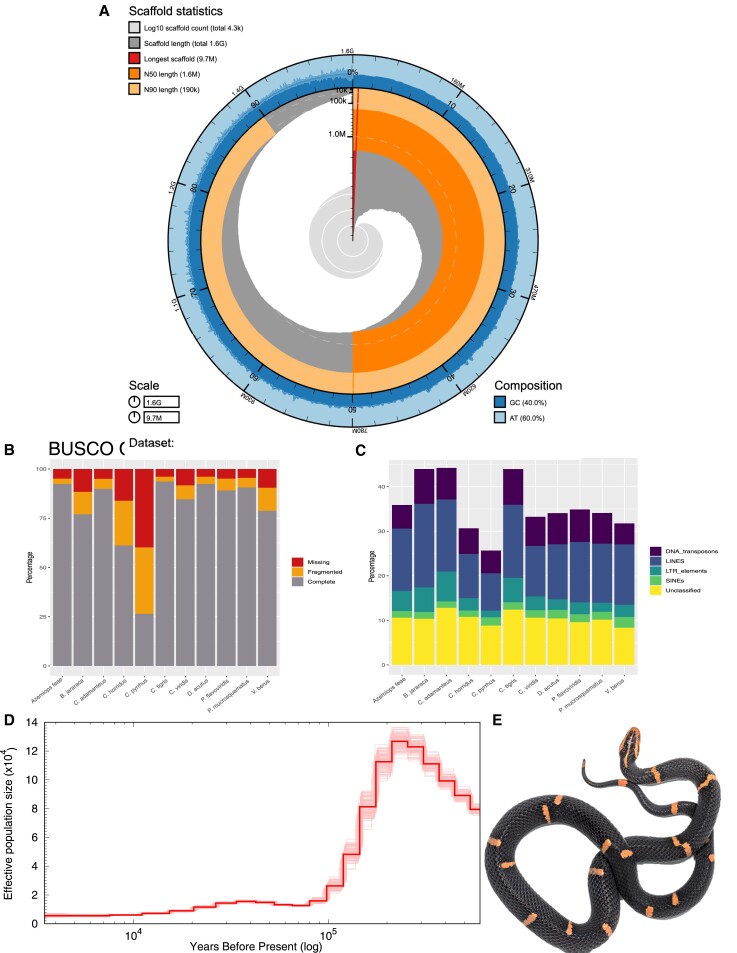
(*A*) Snail plot visualization summarizing metrics of the *Azemiops feae* genome including the length of the longest contig (∼9.7 Mb; red line), N50 (1.59 Mb; dark orange), N90 (190 Kb; light orange), and base composition; (*B*) BUSCO completeness comparing the assembled *Azemiops feae* genome to all published Viperidae genomes. Current phylogenetic relationships within Viperidae are (Viperinae [*V. berus*], Azemiopinae [*A. feae*], Crotalinae [all others]); (*C*) repeat content comparison of major classes for repeat elements across all published Viperidae genomes; (*D*) demographic history of *A. feae* using PSMC, shaded lines represent 100 bootstrap estimates demonstrating that this taxon has had a low, stable effective population size for the last 100 ka; (*E*) photograph of the *A. feae* specimen sequenced here (photo credit to Danny Goodding).

Sequencing resulted in 4.21 million PacBio Sequel I reads (average read length 7,631 bp, a total of 32.2 gigabases, and ∼20× genome coverage) and 665 million 250 bp PE Illumina reads (∼104× genome coverage). Using MaSurCa v3.2.8 (Zimin et al. 2013), we estimated the genome size for *A. feae* as 1.56 Gb, similar to other snakes (supplementary table S1, Supplementary Material online). The final hybrid assembly resulted in 4,303 total scaffolds, with an N50 of 1.597 Mb, a maximum contig length of 9.67 Mb, with 97.6% of the genome assembly in contigs >50 Kb (fig. 1*A*). Kraken (Wood and Salzberg 2014) identified 17 scaffolds of potential bacterial contamination, however, BLAST results of these scaffolds identified them as eukaryotic sequence, often as repetitive sequence from snakes.

We recovered 3,101 (92.4%) complete and 89 (2.7%) fragmented BUSCO loci (fig. 1*B*). Using MAKER v2.31.8, a total of 13,229 protein-coding genes were annotated throughout the genome. Repeat masking indicated that 37.8% of the assembled genome consisted of repetitive sequence (fig. 1*C*). These repetitive sequences were primarily composed of long interspersed nuclear elements (LINEs; 14% of the total genome assembly), unclassified repetitive sequences (10.6%), and DNA transposons (5.3%). We find that the total repeat content of *A. feae* falls within the range of other viperid genomes publicly available, where repetitive element content ranges from 27.5% to 46.7% (fig. 1*C*). Furthermore, repetitive elements within the LINEs families are abundant and recently active in squamates when compared with mammals and birds (Pasquesi et al. 2018). An abundance of LINE repetitive elements is also observed in *A. feae* where chicken repeat 1, Bovine-B, and L2 LINEs together account for ∼10% of the genome.

### Venom Evolution

**Fig. 2.**
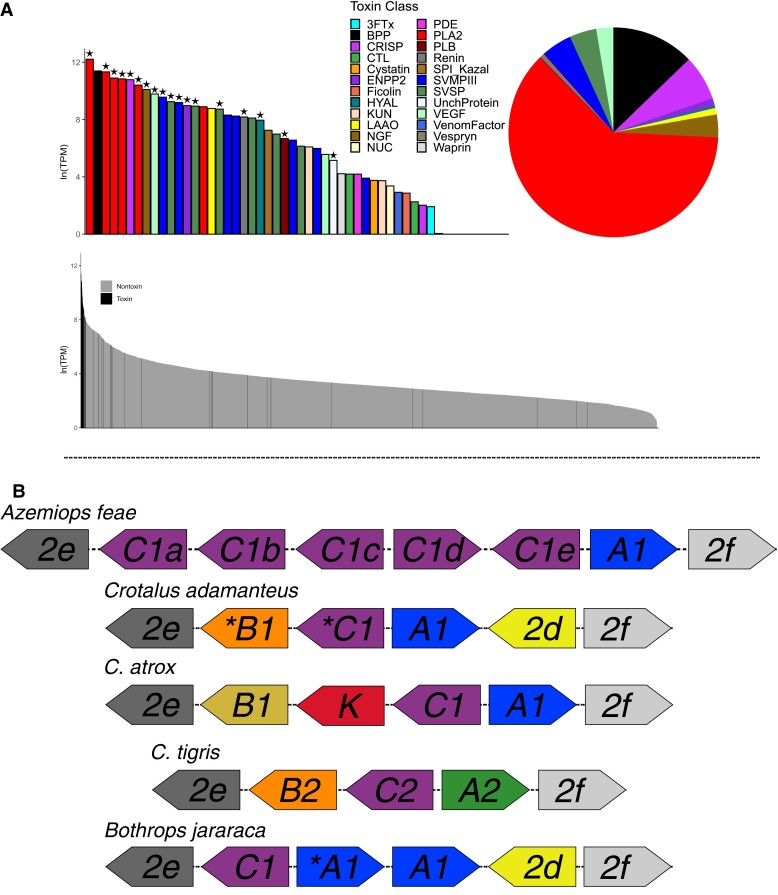
(*A*) The expression of each recovered toxin transcript plotted as ln(TPM) and colored by toxin class, a ‘*’ above a toxin indicates verification via proteomics. The pie-chart represents the proportion of toxin gene expression by class, demonstrating the large proportion of PLA_2_ gene expression within *A. feae*. Gray and black histogram represents total toxin and nontoxin gene expression within the venom gland; (*B*) schematic architecture of the PLA_2_ gene family in *A. feae* compared with members of the Crotalinae (*Crotalus* species and *Bothrops jararaca*) demonstrating the shared architecture of PLA_2_-gC::gA1 across taxa. This also illustrates the numerous hypothesized independent duplications of PLA_2_-gCs within *A. feae* and gene losses in members of the Viperidae. Toxin abbreviations: three-finger toxin (3FTx), Bradykinin-potentiating peptides (BPP), cysteine-rich secretory proteins (CRISP), C-type lectins (CTL), ectonucleotide pyrophosphatase/phosphodiesterase 2 (ENPP2), hyaluronidase (HYAL), Kunitz-type proteinase inhibitor (KUN), l-amino acid oxidase (LAAO), nerve growth factor (NGF), Ecto 5′ nucleotidase (NUC), phosphodiesterase (PDE), phospholipase A_2_ (PLA_2_), phospholipase B (PLB), Kazal-type serine protease inhibitor (SPI_Kazal), snake venom metalloproteinase (SVMP), snake-venom serine protease (SVSP), uncharacterized protein (UnchProtein), and vascular endothelial growth factor (VEGF).

The venom gland transcriptome assembly and annotation resulted in 3,020 nonredundant nontoxin and 40 nonredundant toxin transcripts (fig. 2*A*). These annotated toxins accounted for 69.6% of the total transcriptome expression. Using mass spectrometry (MS), we generated proteomic data to confirm the presence of 18 (45%) of the toxin transcripts in the venom. Using the venom gland transcriptome data, we identified and annotated a total of 51 genes encoding toxic proteins across 30 genomic scaffolds (supplementary table S2, Supplementary Material online).

The venom gland transcriptome of *A. feae* was dominated by six PLA_2_s, a bradykinin-potentiating peptide (azemiopsin; Utkin et al. 2012), and a cysteine-rich secretory protein (CRISP) (fig. 2*A*; supplementary table S2, Supplementary Material online). PLA_2_s accounted for 61.9% of toxin expression (fig. 2*A*). Previous studies have found that PLA_2_s are the most dominant toxin in *Azemiops* venom gland transcriptomes, however, the frequencies of other toxin components differ between our study and published *Azemiops* venom gland transcriptomes (Babenko et al. 2020). Variation in venom composition within populations and between species is commonly documented in snakes (Schenberg 1959; Glenn and Straight 1989); additional sampling and studies are necessary to fully understand the variation present in *Azemiops* venom composition as well as the molecular mechanisms underlying this variation.

Proteomic analyses confirmed the presence of most toxin classes identified in the transcriptomic data. For example, many of the SVMPs, CRISP, snake-venom nerve growth factor (NGF), vascular endothelial growth factor (VEGF), and renin were confirmed via quantitative MS along with most of the PLA_2_s (fig. 2*A*). Many toxins not verified had low expression levels, but this also included the highly expressed azemiopsin and several snake-venom metalloproteinase III toxins. There is a high correlation between venom gland transcriptomes and proteomes and a failure to detect putative toxins proteomically is likely a result of the misassignment of mRNA as toxins or because of proteomic detection thresholds (Rokyta et al. 2015). We suggest the reason some highly expressed toxins in the transcriptome were not verified with MS is because of posttranslational modifications that reduce the active toxins to very small peptides (e.g., note that the BPP toxin was not confirmed here because the <14mers of BPPs are below the threshold of detection using MS; Sciani and Pimenta 2017), resulting in false-negative results.

**Fig. 3.**
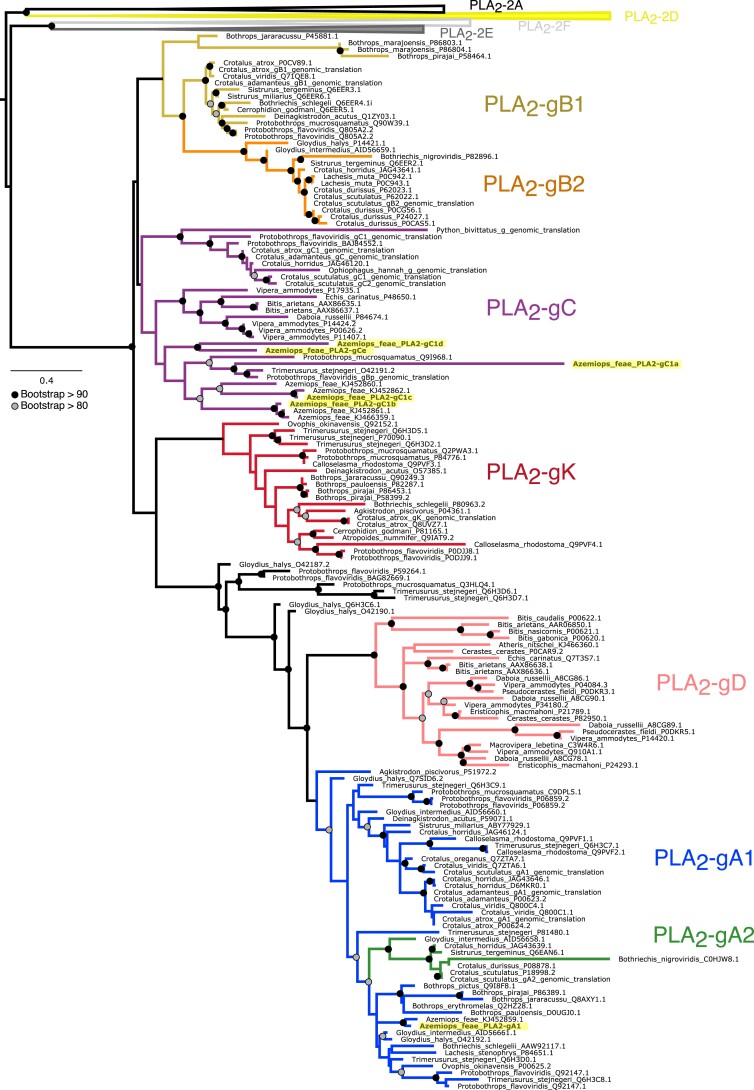
Maximum-likelihood protein phylogeny of PLA_2_g2 proteins sampled broadly across the Viperidae. Different colors represent named classes of PLA_2_g2 proteins. Newly sequenced *A. feae* PLA_2_s are in bold and highlighted in yellow, five of these PLA_2_ proteins cluster within the PLA_2_-gC clade, whereas one is nested within the PLA_2_-gA1 clade. Black circles represent >90% bootstrap support, gray circles represent >80% BS. All terminals include Genbank accession numbers, those listed as “genomic translation” are from Dowell et al. (2016).

We identified six distinct toxin PLA_2_ genes present in the genome, transcriptome, and confirmed five of these via MS (PLA_2_-gC1a was not confirmed; fig. 2*C*). Each of these PLA_2_ genes had transcript per million values >42,000 inferred using StringTie (supplementary table S3, Supplementary Material online). This is in contrast to previous work that identified several PLA_2_ isoforms, only a few of which were thought to be expressed in the venom gland (Tsai et al. 2016; Babenko et al. 2020). It is possible that differences in the number of PLA_2_ genes identified between these studies and here could reflect gene copy number polymorphism within *A. feae*. The proliferation of the PLA_2_ gene family has gained interest because of its prominent role in venom within both elapids and viperids as two distinct expansion events (Lynch 2007). All six *Azemiops* PLA_2_s were tandemly repeated between two nonvenom expressed PLA_2_ genes (PLA_2_-g2e and PLA_2_-g2f), flanked by the OTUD3 and MUL1 genes, a conserved pattern across tetrapods (Dowell et al. 2016). To classify the sequenced PLA_2_s, we combined our data with publicly available sequences and reconstructed a phylogeny based on amino acid-translated sequences (fig. 3). Five of the six *Azemiops* PLA_2_s clustered with the PLA_2_-gC group. It has been hypothesized that this group is ancestral to the pitviper PLA_2_ gene family expansion, which many true vipers and pitvipers possess (this gene is also present in *Ophiophagus* and *Python*; fig. 3; Dowell et al. 2016). The high number of PLA_2_-gCs likely represents *Azemiops* lineage-specific gene duplications leading to novel venom proteins. PLA_2_ gene duplications with subsequent neofunctionalization resulting in increasingly complex venom composition are well documented (Kini 2005; Lynch 2007). The sixth PLA_2_, along with a previously published *Azemiops* PLA_2_ (Tsai et al. 2016), clustered with the PLA_2_-gA1 group (fig. 3). The gC::gA1 PLA2s are the only two genes that are shared across several pitviper taxa (Dowell et al. 2018; Almeida et al. 2021) and this pair of PLA_2_ genes would have been present in the ancestor of *Azemiops* and pitvipers. Additional PLA_2_ genes are shared between viperids and other tetrapods, for example, the PLA_2_-2d gene in *C. adamanteus* and *Bothrops jararaca* (PLA_2_GD; fig. 2*B*) suggests that this gene has an ancient origin. However, its absence in several other *Crotalus* species and *Azemiops*, suggests that PLA_2_ genes are frequently lost. Overall, this supports the notion that gene loss and lineage-specific duplications together lead to a diversity of toxins expressed in snake venoms (Rokyta et al. 2013; Dowell et al. 2016; Mason et al. 2020).

Renin was located on a genomic scaffold with several SVMP genes, expressed in the venom gland transcriptome, and confirmed using proteomic analyses to be present in the venom (fig. 2*A*). Previous analysis of *Azemiops* venom has also identified the presence of renin in the transcriptome and proteome (Babenko et al. 2020). Furthermore, renin has been found in the venom of *Echis* and hypothesized to have toxic properties including inducing local hypertension in envenomated prey, thereby exacerbating tissue disruption by other toxins in the venom (Wagstaff and Harrison 2006). We tested whether this gene was expressed in other tissues of *Azemiops* and found that, whereas renin made up to 0.22% of the total venom gland transcriptome, it composed only 0.02% of the blood transcriptome and was not detected in any other tissue. We suggest that renin has been coopted as a venom protein within viperids and may be found to be more widespread taxonomically than currently recognized.

### Demographic History

Climate has fluctuated greatly throughout the Quaternary, influencing the geographic distributions and population sizes of species globally (Hewitt 2000). However, the extent of these climatic changes influencing population size change in East Asia is less well known; codistributed species have been reported as having stable population sizes, population expansions, or declining population sizes through time (Yan et al. 2013; Qu et al. 2015; Guo et al. 2016). The results from our PSMC analysis demonstrate that *A. feae* effective population size has been relatively stable over the past 100,000 years, suggesting glacial cycles of the Quaternary have had little influence on the demography of this taxon (fig. 1*D*). These results are consistent with the East Asian mountains being climatically stable throughout glacial cycles allowing for effective population size persistence for many species (Qu et al. 2015). Future comparative population genomic studies that take a trait-based approach can illuminate community-level demographic processes across this region (Provost et al. 2021).

## Materials and Methods

### Sample Preparation

A single adult female was acquired from the pet-trade for this study. Venom was collected, vacuum dehydrated, and stored at −20 °C. The snake was euthanized 4 days after venom collection with MS-222 (Beaupre et al. 2004) and the specimen was accessioned at the Florida Museum of Natural History (UF:Herp:192944). DNA was extracted using a standard phenol–chloroform–isoamyl protocol. Genomic libraries were prepared with the TruSeq DNA PCR-Free library prep kit and two lanes of Illumina HiSeq PE250 genomic reads were generated at Florida State University. High molecular weight (HMW) DNA was sent to the University of Delaware Core for PacBio library prep and sequenced using six cells on the Sequel I. See Supplementary material online for details on morphology and sample prep.

Total RNA was extracted from 12 tissues (see Supplementary material online) using a standard TRIzol method (Rokyta et al. 2012). mRNA was isolated from 1,000 ng total RNA using the NEB-Next Poly(A) mRNA magnetic isolation kit and cDNA library preparation was performed using NEB-Next Ultra RNA Library Prep Kit (New England Biolabs) following the manufacturer’s protocols. Libraries were sequenced on an Illumina HiSeq PE250.

### Genome Assembly and Annotation

Illumina reads were first trimmed using Trim Galore! (https://github.com/FelixKrueger/TrimGalore) with default settings. Hybrid de novo genome assembly was performed on the PacBio continuous long reads data and Illumina short-read data using MaSurCa v3.2.8 (Zimin et al. 2013) with default settings. Bacterial contamination in the assembly was assessed using Kraken v2.0 (Wood and Salzberg 2014).

We annotated repeat elements using RepeatModeler and RepeatMasker (Smit et al. 2015; Flynn et al. 2020). Using MAKER v2.31.8 (Holt and Yandell 2011), we annotated coding sequences using the filtered, assembled transcripts, species-specific repeat library, and published protein-coding genes. Following this initial run, we used BUSCO and the genome assembly to train AUGUSTUS (Stanke et al. 2006) with three iterations. See the Supplementary material online for details on genome annotation. We downloaded all published Viperidae genomes and ran RepeatModeler and RepeatMasker v4.1.1 to identify the total percent of each genome that consists of repetitive elements. For each of these viperid genomes, we ran BUSCO v4.1.4 with the vertebrate gene set to assess completeness.

### Transcriptomics and Proteomics

Reads from all transcriptomes were trimmed using Trim Galore! and were assembled using several de novo methods then combined (Holding et al. 2018). The assembled venom gland contigs were annotated via blastx (v. 2.2.31+) searches against the UniProt database. Toxins were parsed from “nontoxin” sequences and coding regions were annotated by clustering sequences using cd-hit-est to a known database of annotated snake toxins (Rokyta et al. 2012, 2013, 2015, 2017). Additional toxin contigs were manually annotated by comparing sequences to the blastx results. After genome annotation, we used StringTie (Pertea et al. 2015) to estimate transcript expression. See Supplementary material online for details on transcriptomic and proteomic analyses.

### Venom Evolution

To investigate the evolutionary history of the PLA_2_ gene family within *Azemiops*, we downloaded the PLA_2_ protein dataset used in Dowell et al. (2016) and Tsai et al. (2016). These amino acid sequences were aligned with the PLA_2_s sequenced here and identified as toxins using muscle (Edgar 2004). A maximum-likelihood gene-tree was inferred with 1,000 bootstrap replicates to assess node support in IQtree v1.6.10 (Hoang et al. 2018).

Because renin was present in both the venom gland transcriptome and MS analysis, we tested whether this gene is expressed elsewhere in the body. We measured relative expression of this gene across the combined venom glands and the other tissues sequenced using RSEM v1.3.0 (Li and Dewey 2011) with default Bowtie2 settings.

### Demographic History

To assess historical changes in *N_e_*, we used the Pairwise Sequentially Markovian Coalescent (PSMC; Li and Durbin 2011). This method infers *N_e_* and identifies recombination events from a single diploid genome sequence using a hidden Markov model. Using coalescent theory, PSMC models pairwise sequence divergence as proportional to the time of coalescence, and where the rate of coalescence in a time period is inversely proportional to *N_e_*. We used samtools (Li et al. 2009) following authors’ recommendations to generate a diploid consensus sequence (https://github.com/lh3/psmc). PSMC was run with default settings and 100 bootstrap replicates. This analysis was scaled assuming a genome-wide mutation rate of 2 × 10^−8^ per site per year (Harrington et al. 2017) and a generation time of 3 years.

## Supplementary Material

evac082_Supplementary_Data

## Data Availability

Data presented in this article are available from NCBI under BioProject PRJNA817186, BioSample SAMN26749269, and the SRA: SAMN26749269 (Illumina genomic data); SAMN26749269 (PacBio genomic data); SAMN26749269 (Illumina RNAseq data).
