# Correction to: De Novo Genome Assembly Highlights the Role of Lineage-Specific Gene Duplications in the Evolution of Venom in Fea's Viper (*Azemiops feae*)

**DOI:** 10.1093/gbe/evaf033

**Published:** 2025-03-14

This is a correction to: Edward A Myers *et al.*, De Novo Genome Assembly Highlights the Role of Lineage-Specific Gene Duplications in the Evolution of Venom in Fea's Viper (*Azemiops feae*), *Genome Biology and Evolution*, Volume 14, Issue 7, July 2022, https://doi.org/10.1093/gbe/evac082

In the published version of this article, grant funding from the National Institutes of Health was incorrectly listed as: “P20GM139767”. The grant ID should read: “P20GM139769”.

The error is outlined only in this notice to preserve the version of record.

