# Supplementary material for: De Novo Genome Assembly Highlights the Role of Lineage-Specific Gene Duplications in the Evolution of Venom in Fea's Viper (Azemiops feae)
Source: Genome Biol Evol. 2022 Jun 7;14(7):evac082. doi: 10.1093/gbe/evac082 (PMC9256536; doi:10.1093/gbe/evac082)
Supplement: evac082_Supplementary_Data [file evac082_supplementary_data.zip › Azemiops_Supplemental_Methods_GBE_R1.docx]

**Supplemental Materials and Methods**

*Specimen Identification*

The wild-caught specimen we obtained and sequenced here is of unknown geographic origin and the collection locality could not be restricted *post-hoc* due to the limited amount of geo-referenced genetic and morphological resources available for comparison. Furthermore, the genus *Azemiops* has recently been the subject of unresolved taxonomic revision. In 2013, *Azemiops feae* sensu lato was split into two species, *A. feae* ­sensu stricto and *A. kharini*, separated by the Red River in East Asia. This taxonomic decision was based on geography, coloration, morphology, and osteology (Orlov et al. 2013). Futhermore, the authors only examined 25 (13 *A. feae* and 12 *A. kharini*) total specimens from a small portion of the known geographic distribution of *Azemiops* and did not analyze any molecular data. However, a recent phylogenetic study using 96 molecular markers recovered *A. kharini* as polyphyletic, nested within *A. feae*, casting doubt on the taxonomic validity of *A. kharini* (Li et al. 2020). Given these uncertainties, some authors have refrained from using the newly proposed taxonomy (Tsai et al. 2016, 2021; Babenko et al. 2020). However, pending more thorough taxonomic investigations and an examination of the sequenced specimens, we acknowledge the taxonomy of *Azemiops* remains unresolved.

We attempted to diagnose the specimen sequenced here according to Orlov et al. (2013) and describe its external morphological features. After each character, we denote whether the value falls within the known variation of *A. feae* [f], *A. kharini* [k], or both species [fk]. General description: head coloration predominately black with a pale longitudinal stripe [f]; transverse semi-bands on body 11R, 10L (of which the first anterior 3 merge); transverse bands on tail 3; sex ♀; mass 57.55 g; snout-vent length 588 mm; and total length 672 mm. Scalation as follows: ventral scales 181 [f]; pre-ventrals 1; subcaudals 39 [f]; dorsal scale row formula 17/17/15 [k]; supralabials 6R (3^rd^ entering orbit), 6L (3^rd^ entering orbit) [fk]; infralabials 8R, 8L; temporals 2+2R, 2+2L [f]; preoculars 3R, 3L [fk]; postoculars 2R, 2L [fk]; suboculars 0R, 0L [fk]; and loreal present R, L [fk]. Based on these characters and the diagnosis by Orlov et al. (2013), our specimen displays features of both species and exhibits some unknown variation. Thus, we tentatively identify our specimen as *Azemiops feae*. The voucher specimen (Field Number: CLP 2232) is accessioned at the Florida Museum of Natural History under the catalogue number UF:Herp:192944 for future reference and taxonomic evaluation.

*Sample Preparation*

Venom was collected by allowing the snake to bite a parafilm-sealed sterile collection cup. Venom was then vacuum dehydrated and stored at -20C for proteomics. The snake was euthanized four days after venom collection with MS-222 following ASIH protocols (Beaupre et al. 2004) under Animal Care and Use Protocol: University of Central Florida IACUC 13–17 W. Tissues were immediately dissected and placed in RNA-later, flash frozen at -80C, or placed in TRIzol solution for immediate RNA extraction; the specimen has been accessioned at the Florida Museum of Natural History (specimen no. UF:Herp:192944). DNA for short read libraries was extracted from blood stored in ethanol using a standard phenol-chloroform-isoamyl extraction protocol. Genomic libraries were prepared with the TruSeq DNA PCR-Free library prep kit following the manufacturer’s protocol. Two lanes of Illumina HiSeq PE250 shotgun genomic reads were generated at the Translational Science Laboratory in the College of Medicine at Florida State University. HMW DNA was sent to the University of Delaware Core for PacBio library prep and sequenced using six cells on the Sequel I platform.

Total RNA was extracted from 12 individual tissues (blood, cloaca, gallbladder, heart, liver, lung, muscle, pancreas, small intestine, stomach, and left and right venom glands) using a standard TRIzol method (Rokyta et al. 2012). mRNA was isolated from 1000 ng total RNA using the NEBNext Poly(A) mRNA magnetic isolation kit (New England Biolabs) and cDNA library preparation was immediately performed using NEB-Next Ultra RNA Library Prep Kit for Illumina (New England Biolabs) following the manufacturer’s protocols. Library quality was checked using an Agilent 2100 Bioanalyzer and concentrations checked via KAPA qPCR. Libraries were sequenced on an Illumina HiSeq at the Translational Science Laboratory in the College of Medicine at FSU.

*Genome Assembly and Annotation*

Illumina reads were first trimmed using Trim Galore! (https://github.com/FelixKrueger/TrimGalore) with default settings. Hybrid *de novo* genome assembly was performed on the PacBio continuous long reads (CLR) data and Illumina short read data using MaSurCa v3.2.8 (Zimin et al. 2013) with default settings on the Clemson University Palmetto Cluster. To check for bacterial contamination in the assembly we used Kraken v2.0 with the standard database (Wood & Salzberg 2014), we then used BLAST to further identify all scaffolds flagged as potential contamination by Kraken.

We annotated repeat elements in the *de novo* assembly using RepeatModeler and RepeatMasker (Smit et al. 2015; Flynn et al. 2020). Then, using MAKER v2.31.8 (Holt & Yandell 2011) we annotated coding sequences using the filtered, *de novo* assembled transcripts, the species-specific repeat library, and all protein-coding genes from *Anolis carolinensis* (Alföldi et al. 2011) and *Ophiophagus hannah* (Vonk et al. 2013). Following the initial MAKER run, we used BUSCO v3 and the *de novo* genome assembly to train AUGUSTUS (Stanke et al. 2006) with three iterations. After the final iteration, gene IDs were ascribed based on homology as described in Schield et al. (2019). Briefly, we used blastp with an e-value of 1e-6 to search a UniProt database containing all protein-coding genes from *A. carolinensis*, *O. hannah*, and *Crotalus viridis*. Additional annotations of venom-gene families were performed using a combination of empirical annotation in FGENESH+ (Solovyev et al. 2006) as described in Dowell et al. (2018) and Schield et al. (2019) as well as manual annotation using the RNA-seq alignments in HiSat2 v2.1.0 (Kim et al. 2015).

To compare assembly completeness and repeat element content, we downloaded all published Viperidae genomes available from NCBI and GigaScience Database (*Bothrops jararaca* [Genbank assembly GCA_018340635.1], *Crotalus adamanteus* [GCA_018446365.1], *C. horridus* [GCA_001625485.1], *C. pyrrhus* [GCA_000737285.1], *C. tigris* [GCA_016545835.1], *C. viridis* [GCA_001625485.1], *Deinagkistrodon acutus* [GigaScience accession <http://dx.doi.org/10.5524/100196>], *Protobothrops flavoviridis* [GCA_003402635.1], *P. mucrosquamatus* [GCA_001527695.3], *Vipera berus* [GCA_000800605.1]; Almeida et al. 2021; Hogan et al. 2021; Gilbert et al. 2014; Margres et al. 2021; Schield et al. 2019; Yin et al. 2016; Shibata et al. 2018; Aird et al. 2017). For each species we ran RepeatModeler to construct species specific repeat element databases and then ran RepeatMasker v4.1.1 to identify the total percent of the genome that consists of repetitive elements. Additionally, for each of these viperid genome assemblies we ran BUSCO v4.1.4 vertebrate gene set to assess completeness.

*Transcriptomics and Proteomics*

Reads from the 12 individual transcriptomes were quality trimmed using Trim Galore! with a minimum phred score of 5 and minimum read length of 75 bp. Transcriptomes were *de novo* assembled using Extender (Rokyta et al. 2012), SeqMan NGen (Lasergene DNAStar; Madison, WI: https://www.dnastar.com/software/lasergene/seqman-ngen/), and Trinity (Grabherr et al. 2011) and combined to yield as many full-length transcripts as possible (Holding et al. 2018). Left and right venom glands were used for venom gene annotation and the ten other tissues were used for genome annotation by AUGUSTUS.

The assembled venom gland contigs were annotated via blastx (v. 2.2.31+) searches against the UniProt database with a minimum e-value of 10^−4^ and percent identity ≥ 90%. Toxins were parsed from “nontoxin” sequences and coding regions for both toxins and “nontoxins” were annotated by clustering sequences using cd-hit-est to a known database of previously annotated snake toxins and other protein sequences (Rokyta et al. 2013, 2012, 2015, 2017). Sequences and associated signal peptides with a match percentage of 80% were automatically annotated. Toxin contigs which were not automatically annotated were manually annotated by comparing the sequences to the blastx results. All fragmented and chimeric transcripts were removed by mapping reads to the annotated transcriptome and transcripts with zero coverage at any position were removed (github.com/masonaj157/ChimeraKiller). Chimeric transcripts were then reported by searching for a difference >75% (-d 0.75) in the average length of reads on either side of a given site based on the average read size. The remaining transcripts were clustered with a sequence identity threshold of 98% using cd-hit to reduce redundancy of repeat transcripts and cluster allelic variation at single loci. The final annotated transcriptome contained 128,017 transcripts. After using the annotated transcriptome to facilitated genome annotation, we used StringTie (Pertea et al. 2015) to estimate transcript expression on the *de novo* assembled genome.

For MS analyses we digested approximately 5 μg of venom using the Calbiochem ProteoExtract All-in-One Trypsin Digestion Kit (Merck, Darmstadt, Germany) according to the manufacturer’s instructions and using LC/MS grade solvents. Samples were dried in a SpeedVac at 25 °C for 1 hour and stored at −20 °C until use. To initiate the mass spectrometry run, the resulting dried and digested tryptic peptides were resuspended in 0.1% formic acid at a final concentration of 250 ng/μL. Three digested *E. coli* proteins-purchased from Abcam at known concentrations and mixed in the specified proportions (1000×) prior to digestion-were used as internal standards: 25 fmol of P00811 (Beta-lactamase ampC), 250 fmol of P31658 (Protein deglycase 1), and 2500 fmol of P31697 (Chaperone protein FimC) per injection. Final sample concentrations were achieved by infusing the internal standard peptide mix into samples. For the LCMS/MS run, a 2 μL aliquot was analyzed using an externally calibrated Thermo Q Exactive HF (high-resolution electrospray tandem mass spectrometer) in conjunction with Dionex UltiMate3000 RSLCnano System. A 2 μL sample was aspirated into a 50 μL loop and loaded onto the trap column (Thermo μ-Precolumn 5 mm, with nanoViper tubing 30 μm i.d. ×10 cm). For separation on the analytical column (Acclaim pepmap RSLC 75 μMx 15 cm nanoviper), the flow rate was set to 300 nl/min. Mobile phase A was composed of 99.9% H2O (EMD Omni Solvent) and 0.1% formic acid, and mobile phase B was composed of 99.9% ACN and 0.1% formic acid. We performed a 60 min linear gradient from 3% to 45% B. The LC eluent was directly nanosprayed into the Q Exactive HF mass spectrometer (Thermo Scientific), and during the chromatographic separation, the Q Exactive HF was operated in a data-dependent mode and under direct control of the Thermo Excalibur 3.1.66 (Thermo Scientific). Resulting MS data were acquired using a data-dependent top-20 method for the Q Exactive HF platform, dynamically choosing the most abundant not-yet-sequenced precursor ions from the survey scans (350–1700).

Sequencing was performed via higher energy collisional dissociation fragmentation with a target value of 105 ions determined with predictive automatic gain control. Full scans (350–1700 m/z) were performed at 60,000 resolution in profile mode. MS2 were acquired in centroid mode at 15,000 resolution. We excluded ions with a single charge, charges more than seven, or unassigned charge. A 15-s dynamic exclusion window was used. We searched the resulting raw files with Proteome Discoverer 2.2 using SequestHT as the search engine with custom-generated FASTA databases and percolator as peptide validator. The SequestHT search parameters used were: enzyme name = Trypsin, maximum missed cleavage = 2, minimum peptide length = 6, maximum peptide length = 144, maximum delta Cn = 0.05, precursor mass tolerance = 10 ppm, fragment mass tolerance = 0.2 Da, dynamic modifications, carbamidomethyl+ 57.021 Da(C) and oxidation + 15.995 Da(M). Protein identities were validated using Scaffold (version 4.3.4, Proteome Software Inc., Portland, OR, USA) software. We accepted protein identities based on a 1.0% false discovery rate (FDR) using the Scaffold Local FDR algorithm and a minimum of one recognized peptide. We considered a transcript proteomically detected if it was found in at least one of the three replicates.

*Venom Evolution*

We used our newly generated and previously published sequences of PLA_2_ genes to investigate the evolutionary history of this gene family within *Azemiops*. We downloaded the PLA_2_ protein dataset used in (Dowell et al. 2016) to classify *Crotalus* PLA_2_ genes, sequences from Tsai et al. (2016), and combined these with the six PLA_2_s that were expressed in the venom gland and confirmed via MS after translating our sequence data to amino acids. These amino acid sequences were aligned using muscle (Edgar 2004) and a maximum likelihood gene-tree was inferred with 1000 bootstrap replicates to assess node support in IQtree v1.6.10 (Hoang et al. 2018).

Because renin was present in both the venom gland transcriptome and MS analysis, we tested whether this gene is expressed elsewhere in the body or has been potentially co-opted for use as a venom component. We measured relative expression of this gene across the combined left and right venom glands and the 10 other tissues sampled. This was done using RSEM v1.3.0 (Li & Dewey 2011) with default Bowtie2 setting with merged reads from each tissue mapped to the assembled renin gene.

*Demographic History*

To assess historical changes in effective population size (*N_e_*) we used the Pairwise Sequentially Markovian Coalescent (PSMC; Li & Durbin 2011). This method infers *N_e_* and identifies recombination events from a single diploid genome sequence using a hidden Markov model. Using coalescent theory, PSMC models pairwise sequence divergence as proportional to the time of coalescence, and where the rate of coalescence in a time period is inversely proportional to *N_e_*. We used the Pairwise Sequentially Markovian Coalescent (PSMC; Li & Durbin 2011) to assess historical population size change in *A. feae*. We used samtools (Li et al. 2009) following the PSMC authors’ recommendations to generate a diploid consensus sequence (<https://github.com/lh3/psmc>). PSMC was run with default settings with 100 bootstrap replicates to assess results. This analysis was scaled assuming a genome-wide mutation rate of 2 x 10^-8^ per site per year (Harrington et al. 2017) and a generation time of 3 years.

**References:**

Aird SD et al. 2017. Population genomic analysis of a pitviper reveals microevolutionary forces underlying venom chemistry. Genome Biol. Evol. 9:2640–2649.

Alföldi J et al. 2011. The genome of the green anole lizard and a comparative analysis with birds and mammals. Nature. 477:587–591.

Almeida DD et al. 2021. Tracking the recruitment and evolution of snake toxins using the evolutionary context provided by the Bothrops jararaca genome. Proc. Natl. Acad. Sci. 118.

Babenko V V et al. 2020. Novel bradykinin-potentiating peptides and three-finger toxins from viper venom: Combined NGS venom gland transcriptomics and quantitative venom proteomics of the Azemiops feae viper. Biomedicines. 8:249.

Beaupre SJ, Jacobson ER, Lillywhite HB, Zamudio K. 2004. Guidelines for use of live amphibians and reptiles in field and laboratory research.

Dowell N et al. 2016. The deep origin and recent loss of venom toxin genes in rattlesnakes. Curr. Biol. 26:2434–2445.

Dowell NL et al. 2018. Extremely divergent haplotypes in two toxin gene complexes encode alternative venom types within rattlesnake species. Curr. Biol. 28:1016–1026.

Edgar RC. 2004. MUSCLE: Multiple sequence alignment with high accuracy and high throughput. Nucleic Acids Res. 32:1792–1797. doi: 10.1093/nar/gkh340.

Flynn JM et al. 2020. RepeatModeler2 for automated genomic discovery of transposable element families. Proc. Natl. Acad. Sci. 117:9451–9457.

Gilbert C et al. 2014. Endogenous hepadnaviruses, bornaviruses and circoviruses in snakes. Proc. R. Soc. London B Biol. Sci. 281:20141122.

Grabherr MG et al. 2011. Trinity: reconstructing a full-length transcriptome without a genome from RNA-Seq data. Nat. Biotechnol. 29:644.

Harrington SM, Hollingsworth BD, Higham TE, Reeder TW. 2017. Pleistocene climatic fluctuations drive isolation and secondary contact in the red diamond rattlesnake (Crotalus ruber) in Baja California. Journal of Biogeography doi: 10.1111/jbi.13114.

Hoang DT, Chernomor O, Von Haeseler A, Minh BQ, Vinh LS. 2018. UFBoot2: improving the ultrafast bootstrap approximation. Mol. Biol. Evol. 35:518–522.

Hogan MP et al. 2021. The Chemosensory Repertoire of the Eastern Diamondback Rattlesnake (Crotalus adamanteus) Reveals Complementary Genetics of Olfactory and Vomeronasal-Type Receptors. J. Mol. Evol. 89:313–328.

Holding ML, Margres MJ, Mason AJ, Parkinson CL, Rokyta DR. 2018. Evaluating the performance of de novo assembly methods for venom-gland transcriptomics. Toxins (Basel). 10:249.

Holt C, Yandell M. 2011. MAKER2: an annotation pipeline and genome-database management tool for second-generation genome projects. BMC Bioinformatics. 12:1–14.

Kim D, Langmead B, Salzberg SL. 2015. HISAT: a fast spliced aligner with low memory requirements. Nat. Methods. 12:357–360.

Li B, Dewey CN. 2011. RSEM: accurate transcript quantification from RNA-Seq data with or without a reference genome. BMC Bioinformatics. 12:1–16.

Li H et al. 2009. The sequence alignment/map format and SAMtools. Bioinformatics. 25:2078–2079.

Li H, Durbin R. 2011. Inference of human population history from individual whole-genome sequences. Nature. 475:493–496.

Li J-N et al. 2020. A large-scale systematic framework of Chinese snakes based on a unified multilocus marker system. Mol. Phylogenet. Evol. 148:106807.

Margres MJ et al. 2021. The Tiger Rattlesnake genome reveals a complex genotype underlying a simple venom phenotype. Proc. Natl. Acad. Sci. U. S. A. 118. doi: 10.1073/pnas.2014634118.

Nikolai LO, Sergei AR, Tao TN. 2013. On the taxonomy and the distribution of snakes of the genus Azemiops Boulenger, 1888: description of a new species. Russ. J. Herpetol. 20:110–128.

Pertea M et al. 2015. StringTie enables improved reconstruction of a transcriptome from RNA-seq reads. Nat. Biotechnol. 33:290–295.

Rokyta DR, Lemmon AR, Margres MJ, Aronow K. 2012. The venom-gland transcriptome of the eastern diamondback rattlesnake (Crotalus adamanteus). BMC Genomics. 13:1–23.

Rokyta DR, Margres MJ, Calvin K. 2015. Post-transcriptional mechanisms contribute little to phenotypic variation in snake venoms. G3 Genes, Genomes, Genet. 5:2375–2382.

Rokyta DR, Margres MJ, Ward MJ, Sanchez EE. 2017. The genetics of venom ontogeny in the eastern diamondback rattlesnake (Crotalus adamanteus). PeerJ. 5:e3249.

Rokyta DR, Wray KP, Margres MJ. 2013. The genesis of an exceptionally lethal venom in the timber rattlesnake (Crotalus horridus) revealed through comparative venom-gland transcriptomics. BMC Genomics. 14:1–21.

Schield DR et al. 2019. The origins and evolution of chromosomes, dosage compensation, and mechanisms underlying venom regulation in snakes. Genome Res. 29:590–601.

Shibata H et al. 2018. The habu genome reveals accelerated evolution of venom protein genes. Sci. Rep. 8:1–11.

Smit AFA, Hubley R, Green P. 2015. RepeatMasker Open-4.0. 2013–2015.

Solovyev V, Kosarev P, Seledsov I, Vorobyev D. 2006. Automatic annotation of eukaryotic genes, pseudogenes and promoters. Genome Biol. 7:1–12.

Stanke M et al. 2006. AUGUSTUS: ab initio prediction of alternative transcripts. Nucleic Acids Res. 34:W435–W439.

Tsai I-H, Wang Y-M, Huang K-F. 2016. Structures of azemiops feae venom phospholipases and cys-rich-secretory protein and implications for taxonomy and toxinology. Toxicon. 114:31–39.

Tsai I-H, Wang Y-M, Lin S-W, Huang K-F. 2021. Structural and bioinformatic analyses of Azemiops venom serine proteases reveal close phylogeographic relationships to pitvipers from eastern China and the New World. Toxicon. 198:93–101.

Vonk FJ et al. 2013. The king cobra genome reveals dynamic gene evolution and adaptation in the snake venom system. Proc. Natl. Acad. Sci. 110:20651–20656.

Wood DE, Salzberg SL. 2014. Kraken: ultrafast metagenomic sequence classification using exact alignments. Genome Biol. 15:1–12.

Yin W et al. 2016. Evolutionary trajectories of snake genes and genomes revealed by comparative analyses of five-pacer viper. Nat. Commun. 7:1–11.

Zimin A V et al. 2013. The MaSuRCA genome assembler. Bioinformatics. 29:2669–2677.
