# Supplementary material for: De Novo Genome Assembly Highlights the Role of Lineage-Specific Gene Duplications in the Evolution of Venom in Fea's Viper (Azemiops feae)
Source: Genome Biol Evol. 2022 Jun 7;14(7):evac082. doi: 10.1093/gbe/evac082 (PMC9256536; doi:10.1093/gbe/evac082)
Supplement: evac082_Supplementary_Data [file evac082_supplementary_data.zip › Supplemental_Materials_R1.docx]

Supplemental Table 1: Summary of genome statistics and completeness of *Azemiops feae* and published viperid genomes.

| **Species** | ***Azemiops feae*** | ***Crotalus pyrrhus*** | ***Crotalus horridus*** | ***Crotalus tigris*** | ***Crotalus adamanteus*** | ***Crotalus viridis*** | ***Deinagkistrodon acutus*** | ***Protobothrops flavoviridis*** | ***Protobothrops mucrosquamatus*** | ***Vipera berus*** | ***Bothrops jararaca*** |
| --- | --- | --- | --- | --- | --- | --- | --- | --- | --- | --- | --- |
|  |  |  |  |  |  |  |  |  |  |  |  |
| **Assembly size (Gb)** | 1.56 | 1.14 | 1.52 | 1.61 | 1.6 | 1.34 | 1.47 | 1.41 | 1.67 | - | 2.1 |
| **Scaffold number** | - | - | - | 160 | - | 7,043 | 162,571 | 84,502 | 52,280 | 28,883 | 27,698 |
| **Scaffold N50, Mb** | - | - | - | 207.72 | - | 179.89 | 2.12 | 0.42 | 0.42 | 0.01 | - |
| **Contig number** | 4,303 | 478,598 | 428,076 | 4,228 | 11,369 | 166,667 | - | 218,011 | 167,851 | 264,195 | 28,752 |
| **Contig N50, Mb** | 1.597 | 0.004 | 0.006 | 2.11 | 0.33 | 0.015 | - | 0.02 | 0.02 | - | 0.16 |
| **GC content (%)** | 39.94 | 38.65 | 34.3 | 39.9 | 39.98 | 38.88 | 39.82 | 38.2 | 40.6 | 41.3 | - |
| **BUSCO completeness (%)** | 92.4 | 26.5 | 61.2 | 93.6 | 89.9 | 84.6 | 92.4 | 89.1 | 90.6 | 78.8 | 92.3 |
| **Sequencing Technology** | PacBio CLR, Illumina PE | Illumina PE | Illumina PE | PacBio CLR, Illumina PE | Illumina PE, Oxford Nanopore, PacBio CLR | Illumina PE, HiC, Chicago | Illumina PE | Illumina PE, Roche 454 | Illumina PE | Illumina PE | PacBio CLR, Illumina PE |

Supplemental Table 2: Diversity of toxin genes found throughout the genome of *Azemiops feae*, as well as their expression and confirmation via mass spectrometry.

| **Gene Family** | **Genes in Genome** | **Genes Expressed** | **qMS Confirmed** | **No. of Scaffolds** |
| --- | --- | --- | --- | --- |
| 3FTx | 1 | 0 | 0 | 1 |
| BPP (Azemiopsin) | 1 | 1 | 0 | 1 |
| CRISP | 2 | 1 | 1 | 1 |
| CTL | 6 | 2 | 0 | 2 |
| Cystatin | 1 | 1 | 0 | 1 |
| ENPP2 | 1 | 1 | 1 | 1 |
| Ficolin | 2 | 1 | 0 | 2 |
| HYAL | 2 | 1 | 1 | 1 |
| KUN | 2 | 2 | 0 | 2 |
| LAAO | 3 | 1 | 0 | 2 |
| NGF | 1 | 1 | 1 | 1 |
| NUC | 1 | 1 | 0 | 1 |
| PDE | 1 | 1 | 0 | 1 |
| PLA2 | 6 | 6 | 5 | 1 |
| PLB | 1 | 1 | 1 | 1 |
| Renin | 1 | 1 | 1 | 1 |
| SVMP-III | 7 (one fragmented) | 7 | 2 | 3 |
| SVSP | 6 | 6 | 3 | 1 |
| Uncharacterized protein | 1 | 1 | 1 | 1 |
| VEGF | 2 | 2 | 1 | 2 |
| Vespryn | 1 | 0 | 0 | 1 |
| VF | 1 | 1 | 0 | 1 |
| Waprin | 1 | 1 | 0 | 1 |

Supplemental Table 3: Expression of the six toxin PLA2 genes in transcript per millions inferred using StringTie.

| **PLA2 Gene** | **TPM** |
| --- | --- |
| PLA_2_-gCa | 42,527 |
| PLA_2_-gCb | 136,826 |
| PLA_2_-gCc | 138,754 |
| PLA_2_-gCd | 86,898 |
| PLA_2_-gCe | 230,916 |
| PLA_2_-gA1 | 361,302 |
